# Risk factors for the development of surgical site infection in bariatric surgery: an integrative review of literature

**DOI:** 10.1590/1518-8345.6309.3798

**Published:** 2023-03-06

**Authors:** Ana Flávia da Silva, Karina Dal Sasso Mendes, Vanessa dos Santos Ribeiro, Cristina Maria Galvão

**Affiliations:** 1 Unimed, Hospital da Unimed, Ribeirão Preto, SP, Brazil; 2 Universidade de São Paulo, Escola de Enfermagem de Ribeirão Preto, PAHO/WHO Collaborating Centre for Nursing Research Development, Ribeirão Preto, SP, Brazil; 3 Scholarship holder at the Conselho Nacional de Desenvolvimento Científico e Tecnológico (CNPq), Brazil.

**Keywords:** Perioperative Nursing, Surgical Wound Infection, Bariatric Surgery, Risk Factors, Perioperative Period, Patient Safety, Enfermagem Perioperatória, Infecção da Ferida Cirúrgica, Cirurgia Bariátrica, Fatores de Risco, Período Perioperatório, Segurança do Paciente, Enfermería Perioperatoria, Infección de la Herida Quirúrgica, Cirugía Bariátrica, Factores de Riesgo, Periodo Perioperatorio, Seguridad del Paciente

## Abstract

**Objective:**

to evaluate evidence on risk factors for the development of surgical site infection in bariatric surgery.

**Method:**

integrative review. The search for primary studies was performed in four databases. The sample consisted of 11 surveys. The methodological quality of the included studies was assessed using tools proposed by the Joanna Briggs Institute. Data analysis and synthesis were performed in a descriptive manner.

**Results:**

surgical site infection rates ranged from 0.4% to 7.6%, considering the results of primary studies, in which patients underwent laparoscopic surgery. In surveys of participants undergoing surgical procedures with different approaches (open, laparoscopic or robotic), infection rates ranged from 0.9% to 12%. Regarding the risk factors for the development of this type of infection, antibiotic prophylaxis, female sex, high Body Mass Index and perioperative hyperglycemia are highlighted.

**Conclusion:**

conducting the integrative review generated a body of evidence that reinforces the importance of implementing effective measures for the prevention and control of surgical site infection, by health professionals, after bariatric surgery, promoting improved care and patient safety in the perioperative period.

## Resumo

**Objetivo:**

avaliar as evidências sobre os fatores de risco para o desenvolvimento de infecção de sítio cirúrgico em cirurgia bariátrica.

**Método:**

revisão integrativa. A busca dos estudos primários foi realizada em quatro bases de dados. A amostra foi composta por 11 pesquisas. A qualidade metodológica dos estudos incluídos foi avaliada por meio de ferramentas propostas pelo *Joanna Briggs Institute*. A análise e a síntese dos dados foram realizadas de maneira descritiva.

**Resultados:**

as taxas de infecção de sítio cirúrgico variaram de 0,4% até 7,6%, considerando os resultados dos estudos primários, cujos pacientes foram submetidos à cirurgia por via laparoscópica. Nas pesquisas com os participantes submetidos aos procedimentos cirúrgicos com diferentes abordagens (aberta, via laparoscópica ou robótica), as taxas de infecção variaram de 0,9% até 12%. Com relação aos fatores de risco para o desenvolvimento deste tipo de infecção, ressaltam-se antibioticoprofilaxia, sexo feminino, Índice de Massa Corporal elevado e hiperglicemia perioperatória.

**Conclusão:**

a condução da revisão gerou corpo de evidências que reforça a importância na implementação de medidas efetivas para prevenção e controle de infecção de sítio cirúrgico pelos profissionais de saúde após cirurgia bariátrica, promovendo a melhoria da assistência e da segurança do paciente no perioperatório.

## Resumen

**Objetivo:**

evaluar las evidencias sobre los factores de riesgo para el desarrollo de infección de sitio quirúrgico en cirugía bariátrica.

**Método:**

revisión integradora. La búsqueda de estudios primarios se realizó en cuatro bases de datos. La muestra estuvo compuesta por 11 investigaciones. La calidad metodológica de los estudios incluidos se evaluó mediante herramientas propuestas por el *Joanna Briggs Institute*. El análisis y la síntesis de los datos se realizaron de manera descriptiva.

**Resultados:**

las tasas de infección del sitio quirúrgico oscilaron entre 0,4% y 7,6%, considerando los resultados de los estudios primarios, en los que los pacientes fueron sometidos a cirugía mediante laparoscopia. En investigaciones con participantes que se sometieron a procedimientos quirúrgicos con diferentes enfoques (abierto, laparoscópico o robótico), las tasas de infección oscilaron entre el 0,9 % y el 12 %. En cuanto a los factores de riesgo para el desarrollo de este tipo de infección, se destacan la profilaxis antibiótica, el sexo femenino, el Índice de Masa Corporal elevado y la hiperglucemia perioperatoria.

**Conclusión:**

la realización de la revisión generó un cuerpo de evidencia que refuerza la importancia de implementar medidas efectivas para la prevención y el control de la infección de sitio quirúrgico, por parte de los profesionales de la salud, después de la cirugía bariátrica, promoviendo la mejora de la atención y la seguridad del paciente en el período perioperatorio.

**Highlights**

(1) Obesity is a public health problem with worldwide repercussions.

(2) Bariatric surgery is a therapeutic option for the treatment of obesity.

(3) Surgical site infection is a relevant complication after bariatric surgery.

(4) Nurses have a relevant role in preventing surgical site infection.

(5) The nurse’s role is essential in the management of complications after bariatric surgery.

## Introduction

Obesity is known as an epidemic disease with worldwide repercussions, recurrent in developed and developing countries, being one of the main risk factors for chronic non-communicable diseases, such as diabetes mellitus and cardiovascular diseases. This disease has multifactorial causes linked to environmental, economic, genetic, metabolic and lifestyle aspects. Through the Body Mass Index (BMI), the World Health Organization defines the diagnosis of obesity, that is, BMI ≥ 30 kg/m^2^ ^1^ ^-^ ^2^.

Bariatric surgery is considered a therapeutic option for the treatment of obesity, when conservative clinical treatment (diet, physical exercises and medication) has not been successful and after careful evaluation by a multidisciplinary team. In addition to the nutritional status and physical conditions of the patient, the team must assess mental health, since the success of the surgery depends on changes in lifestyle, eating habits and the search for emotional balance of the individual who will undergo the procedure. In short, bariatric surgery leads to weight loss, with improvement in body parameters, as well as a reduction in morbidity and mortality associated with obesity^2^ ^-^ ^3^.

Currently, in bariatric surgery, two types of procedures are the most chosen by surgeons, namely, sleeve gastrectomy and Roux-en-Y gastric bypass, which can be performed in open, laparoscopic approaches (minimally invasive surgery) or robotics (robot-assisted surgery)^4^.

Complications after bariatric surgery can be classified as early (during the immediate postoperative period) or late (generally, after 30 days postoperatively). Depending on the type of surgical procedure, early complications include hemorrhage, anastomotic leakage, gastric or small bowel perforation, and deep vein thrombosis/pulmonary embolism, and major late complications are bowel obstruction, gallstone formation, and gastrointestinal hemorrhage^5^.

Surgical site infection (SSI) is also a relevant complication that can affect patients undergoing bariatric surgery, since obesity is a risk factor for the development of this type of infection^6^ ^-^ ^8^. In addition, patients with obesity are more susceptible to developing infectious diseases. However, the mechanisms underlying the increased susceptibility to different types of infections are not well established. Thus, some potential risk factors may be directly involved, including changes in the immune system related to obesity and vitamin D deficiency. Other factors frequently associated with obesity, which do not have a clear cause in the effect relationship, may indirectly favor the appearance or aggravation of infectious diseases. Such factors include changes in respiratory physiology, changes in the skin and soft tissues, comorbidities such as type 2 diabetes mellitus and cardiovascular disease, drug therapy and, above all, underdosing of antimicrobials^8^.

The prevention and early treatment of complications are essential to achieve better results for the patient and, consequently, the success of the therapy. Thus, the performance of a multidisciplinary team is crucial, from the preparation for the surgery and the follow-up in the postoperative period. In this context, the nurse has a prominent role, since this professional is responsible for planning and implementing the necessary nursing care and for health education. In addition, they must have knowledge about complications after bariatric surgery, aimed at their prevention and early detection, effectively helping the patient’s well-being and the new condition of life.

In view of the above, the synthesis of knowledge produced on SSI in bariatric surgery can help the multidisciplinary team, contributing to the improvement of care provided and patient safety, especially nursing care. Thus, the delimited objective of the present review was to evaluate the evidence on the risk factors for the development of surgical site infection in bariatric surgery.

## Method

### Study type

The method of knowledge synthesis selected for conducting this study was the integrative review of the literature (IR). The steps covered were: elaboration of the review question, literature search of primary studies, evaluation of primary studies, data analysis and presentation of the review^9^.

The IR protocol was registered in the *Open Science Framework* (OSF). This platform is open, and the main objective is to support the conduct of research and allow collaboration between researchers, in a global context. The protocol registration took place on April 28, 2021, and the access link was: https://archive.org/details/osf-registrations-fxr6v-v1 and DOI: 10.17605/OSF.IO/FXR6.

### Setting

The IR was carried out in the city of Ribeirão Preto, São Paulo State, Brazil.

### Study period

The study was carried out from March 2021 to March 2022.

### Population

The review question was: “what evidence is available in the literature on risk factors for the development of surgical site infection in bariatric surgery?” To elaborate this question, the acronym PECO was adopted (population with the problem, exposure, comparator and outcome), where P= patient submitted to bariatric surgery; E= risk factors; C= not applicable and O= surgical site infection.

### Selection criteria

Eligibility criteria for the development of IR were: primary studies whose authors investigated risk factors for the development of surgical site infection in bariatric surgery in patients aged ≥ 18 years; published in English, Portuguese, Spanish and from January 2011 to April 2021.

In view of the above, editorial, response letter, secondary studies (eg, systematic review), experience report or expert opinion were excluded from the review sample. The time frame was established to ensure an adequate number of primary studies, since the inclusion of a high volume of research can make it impossible to conduct an integrative review or introduce biases in the following steps of the method.

### Sample definition

Four databases, relevant to the area of health and nursing, were selected for the search for primary studies, namely: PubMed, Cumulative Index to Nursing and Allied Health Literature (CINAHL), Scopus and Latin American and Caribbean Literature in Health Sciences (LILACS).

The three described components of the PECO acronym were used in different combinations of controlled descriptors, keywords and the Boolean operators AND and OR (search strategies for publications in the databases). In two databases (PubMed and Scopus), the controlled descriptors were delimited from the Medical Subject Headings (MeSH) and the search strategies adopted were: “Bariatric Surgery”[Mesh] OR “Bariatric Surgery” OR “Metabolic Surgery” OR “Bariatric Surgical Procedure” OR “Surgical Procedures, Bariatric” OR “Bariatric Surgeries” OR “Roux-en-Y gastric bypass” OR “Sleeve gastrectomy” OR “Weight Loss Surgery” OR “Gastric bypass” OR “Laparoscopic Adjustable Gastric Banding” OR “Duodenal Switch” AND “Surgical Wound Infection”[Mesh] OR “Surgical Wound Infections” OR “Surgical Wound Infection” OR “Surgical Site Infection” OR “Surgical Site Infections” OR “Postoperative Wound Infections” OR “Postoperative Wound Infection” OR “Wound Infections” OR “Wound Infections Surgical” AND “Risk Factors”[Mesh] OR “Risk Factors” OR “Risk Factor”.

In the CINAHL and LILACS databases, the search strategies adopted were similar, but using the base vocabulary (controlled descriptors), that is, CINAHL Headings and Health Science Descriptors (DeCS), respectively. In the databases, the final search strategies for publications were implemented on May 2, 2021.

The EndNote reference manager (version XII - Desktop) was used to remove duplicates of exported results (publications) from the four databases^10^.

The Rayyan platform was used for the selection of primary studies among the reviewers^11^. Thus, this selection was carried out by reading the titles and abstracts of the publications, based on the IR question and the eligibility criteria. This step was performed by two reviewers independently and masked. The masking of the Rayyan platform was open and, in consensus meetings, the reviewers performed the selection of primary studies for full reading. It is noteworthy that in these meetings, a third reviewer assisted in the discussions.

The full reading of the selected primary studies (n=36) was also performed by two reviewers independently. In the event of discrepancies, a third reviewer was consulted to resolve the queries and assist in the final selection of studies included in the IR sample.

In addition to searching the databases, a reviewer manually searched for other research in the references of the primary studies included in the IR, and no new studies were included using this strategy.

The search and selection of primary studies took place from May to July 2021.

### Data collection 

To collect data from the studies included in the review, a script was built with the following items: authors; study title; year of publication; journal name; objective; sample and method detail; statistical analysis; data on the occurrence/incidence of SSI; main results and conclusion. This step was carried out, from August to October 2021, by two reviewers, independently, and through meetings, differences were discussed until consensus.

### Data processing and analysis

The identification of the type of study was according to the name given by the authors of the research included in the review. It should be noted that in two surveys, the necessary information was not found. The studies were called retrospective, since the data were collected from a database entitled Metabolic and Bariatric Surgery Accreditation and Quality Improvement Program (United States of America).

The methodological quality of the primary studies was assessed using tools developed by the Joanna Briggs Institute. This international organization provides free tools for each type of study, that is, randomized clinical trial, quasi-experimental study, cohort study, cross-sectional study, among others. Such tools are composed of questions, and for each one, the reviewer answers yes, no, uncertain or not applicable. Through these questions, the internal validity and risk of bias of the study are evaluated (selection of participants, method adopted and analysis of results)^12^.

The tool entitled JBI Critical Appraisal Checklist for Studies Reporting Prevalence Data was used to evaluate prospective or retrospective studies. For the evaluation of cohort studies, the tool adopted is called JBI Critical Appraisal Checklist for Cohort Studies and, for case-control studies, the tool is called JBI Critical Appraisal Checklist Case Control Studies.

The evaluation of the methodological quality was carried out, in February 2022, by two reviewers, independently, and the divergences were discussed in meetings until consensus.

Data analysis and synthesis were performed in a descriptive manner.

## Results

**Figure 1:**
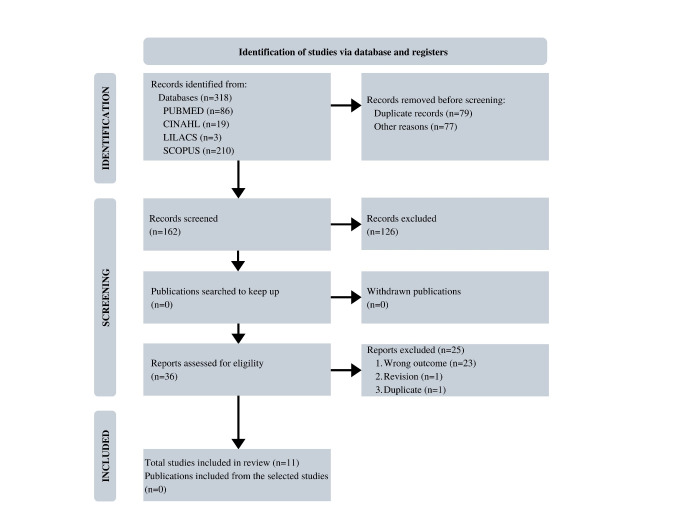
Flowchart of the selection process of primary studies included in the integrative review according to the Preferred Reporting Items for Systematic Review and Meta-Analyses (PRISMA). Ribeirão Preto, SP, Brazil, 2022

In Figure 1, the flowchart of the selection process of the primary studies included in the IR was presented. Thus, of the 318 publications identified in the databases (registries), after applying the eligibility criteria, 36 primary studies were selected for full reading and 11 were part of the review sample.

Fonte: Page, et al.^13^

In Figures 2 and 3, the descriptive synthesis of the primary studies was presented, using the following information: authors and year of publication of the research, sample, type of study, objective(s) and risk factors for the development of SSI. In Figure 2, the primary studies grouped were those in which the target population underwent laparoscopic bariatric surgery.

**Figure 2:** Descriptive synthesis of the primary studies included in the integrative review (laparoscopic surgical approach). Ribeirão Preto, SP, Brazil, 2022

Primary study/year of publication	Type of study/sample	Objective (s)	Risk factors
Ruiz-Tovar, et al. (2013)^14^	Prospective study (authors) Sample: n=40	To investigate the association of comorbidities and pre and postoperative variables with surgical site infection after sleeve gastrectomy.	Preoperative: BMI*>45 kg/m^2^; restrictive disorders identified by pulmonary function tests; serum total protein concentration <5.3 g/dL; plasma cortisol >30 mcg/dL; MCV^†^ < 82 fL. Postoperative: blood glucose > 128 mg/dL; hemoglobin <11 g/dL (variables associated with SSI^‡^ only in univariate analysis)
Lyons, et al. (2014)^15^	Retrospective cohort study (authors) Sample: n=815	To quantify the rate of postoperative infection after bariatric surgery. To determine whether infection-related events contribute to lengthening hospital stays and to assess the effect of risk factors, such as diabetes, on infection rates.	The authors did not analyze risk factors for SSI, but for general infection that occurred in the postoperative period.
Vetter, et al. (2017)^16^	Retrospective study (authors) Sample: n=1,400	To assess whether a secondary, planned wound closure in the upper left abdomen reduces the rate of wound infection and whether such a technique has a positive impact on hospital stay, costs, and postoperative morbidity.	Women; primary wound closure; dyslipidemia and presence of preoperative gastritis (analytical statistical treatment)
Meister, et al. (2018)^17^	Retrospective study (authors) Sample: n=1,981	To assess the significance of perioperative hyperglycemia in different complications of infection (six types of infection were investigated, including surgical site infection) and clinical outcomes.	In univariate analysis, perioperative hyperglycemia was associated with the development of SSI in patients with diabetes.
Dang, et al. (2020)^18^	Retrospective study Sample: n=274,187	To develop a predictive tool for surgical site infection after 30 days of bariatric surgery.	Roux-en-Y gastric bypass surgery; chronic use of steroids or immunosuppressants; women; gastroesophageal reflux disease; hypertension; diabetes mellitus; White breed; long operative time; sleep apnea and high BMI (analytical statistical treatment)
Falvo, et al. (2020)^19^	Retrospective study Sample: n=47,906	To compare short-term results (30 days after surgery), by biological sex, of patients undergoing Roux-en-Y gastric bypass.	In the study there was no analysis of this nature.

* BMI = Body mass index; ^†^MCV = Mean corpuscular volume; ^‡^SSI = Surgical site infection

In Figure 3, patients underwent procedures with different approaches (open, laparoscopic or robotic).

**Figure 3:** Descriptive synthesis of the primary studies included in the integrative review (open, laparoscopic or robotic surgical approach). Ribeirão Preto, SP, Brazil, 2022

Primary study/year of publication	Type of study/sample	Objective (s)	Risk factors
Freeman, et al. (2011)^20^	Prospective cohort study (authors) Sample: n=2,012	To assess the surgical site infection rate of group patients from community hospitals and compare with previously published rates of patients in tertiary hospitals.	Antibiotic prophylaxis (analytical statistical treatment).
Chopra, et al. (2012)^21^	Case-control study (authors) Sample: n=751	To assess the importance of potential risk factors for surgical site infection after bariatric surgery.	Sleep apnea; bipolar disorder; surgery duration (> 180 minutes); use of prophylactic antimicrobials other than cefazolin (analytical statistical treatment).
Gerber, et al. (2018)^22^	Cohort study (authors) Sample: n=47,660	Define the risk of complications and mortality in relation to age after gastric bypass.	Age (analytical statistical treatment).
Ferraz, et al. (2019)^23^	Prospective cohort study (authors) Sample: n=1.596	To present a descriptive analysis of the results of a care package applied to obese patients undergoing bariatric surgery for infection control.	Body mass index; diabetes mellitus (correlation between variables).
Gray, et al. (2020)^4^	Retrospective study (authors) Sample: n=148,260	Explore the routine use of abdominal drain placement in sleeve gastrectomy and Roux-en-Y gastric bypass surgeries to assess associated complications and potential risk factors.	Drain (analytical statistical treatment).

In the methodological evaluation of the primary studies, the tools proposed by the Joanna Briggs Institute were used, and none of them have a scoring system for the general evaluation of the research, but it can be affirmed that a greater quantity of “yes” answers is indicative of better results. methodological quality^24^.

In the evaluation of prospective or retrospective studies (n=6), through the JBI Critical Appraisal Checklist for Studies Reporting Prevalence Data tool, of the nine questions that make up the checklist, in three researches^16^ ^-^ ^18^, eight questions received the answer “yes” in the evaluation carried out by the reviewers and in a study^14^, seven questions received the answer “yes” (Figure 4).

**Figure 4:** Methodological evaluation of primary studies using the JBI Critical Appraisal Checklist for Studies Reporting Prevalence Data tool. Ribeirão Preto, SP, Brazil, 2022

Retrospective/Prospective study	Q1^*^	Q2^†^	Q3^‡^	Q4^§^	Q5^||^	Q6^¶|^	Q7^**^	Q8^††^	Q9^‡‡^	Total (Yes)
Ruiz-Tovar, et al. (2013)^14^	Y^§§^	Y	N^||||^	Y	Y	Y	Y	Y	NA^¶¶^	7
Vetter, et al. (2017)^16^	Y	Y	Y	Y	Y	Y	Y	Y	NA	8
Meister, et al. (2018)^17^	Y	Y	Y	Y	Y	Y	Y	Y	NA	8
Dang, et al. (2020)^18^	Y	Y	Y	Y	Y	Y	Y	Y	NA	8
Falvo, et al. (2020)^19^	Y	Y	Y	Y	Y	U^***^	U	N	NA	5
Gray, et al. (2020)^4^	Y	Y	Y	Y	Y	U	U	Y	NA	6

* Q1 = Is the sample structure appropriate to represent the target population?; ^†^Q2 = Were the study participants appropriately selected?; ^‡^Q3 = Was the sample size adequate?; ^§^Q4 = Were the participants and the study site described in detail?; ^||^Q5 = Was the data analysis performed on a sufficient portion of the identified sample?; ^¶^Q6 = Were valid methods used to identify the condition/disease?; ^**^Q7 = Condition/disease was measured in a standard and reliable way for all participants; ^††^Q8 = Was an appropriate statistical analysis used?; ^‡‡^Q9 = Was the response rate adequate? If the response rate was low, was it managed properly?; ^§§^Y = Yes; ^||||^N = No; ^¶¶^NA =Not applicable; ^***^U = Unclear

The JBI Critical Appraisal Checklist for Cohort Studies tool was used to evaluate the cohort studies (n=4). Of the 11 questions that make up the checklist, in two studies^15^ ^,^ ^22^, seven questions received the answer “yes”, and in the other two^20^ ^,^ ^23^, six questions were also answered “yes” (Figure 5). Using the JBI Critical Appraisal Checklist Case Control Studies tool to evaluate the only case-control study included in the review, the survey received “yes” answers to all checklist questions (10 questions)^21^.

**Figure 5:** Methodological evaluation of primary studies using the JBI Critical Appraisal Checklist for Cohort Studies tool. Ribeirão Preto, SP, Brazil, 2022

Coorth study	Q1^*^	Q2^†^	Q3^‡^	Q4^§^	Q5^||^	Q6^¶|^	Q7^**^	Q8^††^	Q9^‡‡^	Q10^§§^	Q11^||||^	Total (Yes)
Freeman, et al. (2011)^20^	Y^¶|¶|^	Y	Y	N^***^	N	Y	Y	U^†††^	U	U	Y	6
Lyons, et al. (2014)^15^	Y	Y	Y	N	N	Y	Y	Y	Y	NA^‡‡‡^	N	7
Gerber, et al. (2018)^22^	Y	Y	Y	N	N	Y	U	Y	Y	NA	Y	7
Ferraz, et al. (2019)^23^	Y	Y	Y	N	N	Y	U	Y	Y	NA	N	6

* Q1= Were the two groups similar and recruited from the same population?; ^†^Q2 = Were exposures measured similarly to assign participants to exposed and unexposed groups?; ^‡^Q3 = Was exposure validly and reliably measured?; ^§^Q4 = Have confounding factors been identified?; ^||^Q5 = Have the strategies to deal with confounding factors been established?; ^¶^Q6 = Were the groups/participants free of the outcome at baseline (or at the time of exposure)?; ^**^Q7 = Were the results validly and reliably measured?; ^††^Q8 = Was the follow-up time reported long enough for outcomes to occur?; ^‡‡^Q9 = Was the follow-up complete and, if not, were the reasons for losing follow-up described and explored?; ^§§^Q10 = Were strategies used to deal with incomplete follow-up? ^||||^Q11 = Was an appropriate statistical analysis used?; ^¶¶^Y = Yes; ^*** |^N = No; ^†††^U = Unclear; ^‡‡‡^NA = Not applicable

## Discussion

To facilitate the reader’s understanding, the primary studies included in the review were grouped according to the surgical approach. Thus, in six primary studies^14^ ^-^ ^19^, patients underwent laparoscopic bariatric surgery and, in five studies^4^ ^,^ ^20^ ^-^ ^23^, participants underwent procedures with different approaches (open, laparoscopic or robotics).

In the prospective study, the authors investigated the association of comorbidities and variables (pre and postoperatively) with SSI after sleeve gastrectomy. The sample consisted of 40 patients, and SSI was diagnosed in three patients (7.5%), two cases classified as organ/space (intra-abdominal abscess) and one superficial^14^.

In the retrospective cohort study, patients underwent the following types of bariatric surgery: Roux-en-Y gastric bypass, sleeve gastrectomy and adjustable gastric band, with a sample of 815 patients. The incidence of surgery-related infection was 4.2% in the first postoperative month^15^.

In the retrospective study, the sample consisted of 1,400 patients undergoing Roux-en-Y gastric bypass. In this research, a surgical technique was tested (secondary and planned wound closure in the upper left abdomen/typical locus, where the circular stapler for the gastrojejunostomy was inserted into the abdominal cavity), with the aim of reducing infection rates. The overall wound infection rate was 7.6% (n=106) with 9.3% (103/1109) in the primary wound closure group compared to 1.0% (3/291) in the secondary wound closure group (planned and tested) (p <0.001)^16^.

In another retrospective study, the sample consisted of 1,981 patients who underwent Roux-en-Y gastric bypass or sleeve gastrectomy, 38% (n=751) had diabetes and 62% (n=1,230) did not have diabetes. chronic disease. Regarding SSI, in the group of patients without diabetes, the overall rate of superficial SSI was 0.7% (n=9) and organ/space SSI was 0.4% (n=5). In the group of patients with diabetes, the overall rate of superficial SSI was 2.8% (n=21) and of organ/space SSI was 0.9% (n=7)^17^.

In a retrospective study, the authors developed a predictive tool (BariWound) for SSI after 30 days of surgery. Patients undergoing sleeve gastrectomy or Roux-en-Y gastric bypass (n=274,187) were included in the research. Of the investigated sample, 1,841 patients (0.7%) had SSI, 70.1% of which were classified as incisional SSI, 29.0% of organ/space and 0.9% a combination of both types^18^.

In a retrospective study, the sample consisted of patients undergoing Roux-en-Y gastric bypass (n=47,906), who were divided into a male cohort and a female cohort with the same number (n =23,953 each cohort). The female cohort had a higher rate of superficial incisional SSI compared to the male cohort (1.07% versus 0.80%, p=0.002). Regarding organ and space SSI, the infection rate was 0.41% (n=156) in the female cohort and 0.43% (n=104) in the male cohort (no statistically significant difference)^19^.

In view of the above, SSI rates ranged from 0.4% to 7.6%, considering the results of primary studies, in which patients underwent laparoscopic surgery^14^ ^-^ ^19^.

In the literature, in a national cross-sectional study, the authors evaluated the occurrence of complications in bariatric surgery (Roux-en-Y gastric bypass). The sample consisted of 469 patients, and data were collected from medical records and records of outpatient consultations. Participants were followed up for at least one year. The occurrence of postoperative complications that required hospitalization was 24.09% (n=113), with cholecystectomy being the most frequent complication (n=72; 15.35%). Regarding infectious complications, one patient had a superficial abscess (0.21%) and three had a deep abscess (0.63%)^25^.

In this grouping of primary studies included in the review, perioperative hyperglycemia^14^ ^,^ ^17^, female sex^16^ ^,^ ^18^ and high BMI^14^ ^,^ ^18^ were the risk factors investigated in at least two studies and confirmed by means of the statistical treatment used.

In a prospective cohort study, with a sample of 484 patients undergoing abdominal surgery, the defined objectives were to evaluate the independent effect of perioperative hyperglycemia and the incidence of SSI. Most patients underwent cholecystectomy (50.21%), and 0.83% (n=4) underwent bariatric surgery. Of the participants, 18.39% (n=89) had diabetes and 81.61% (n=395) did not have this disease. The incidence of SSI was 20.25% (98/484), with hyperglycemia being an independent risk factor for this type of infection^26^.

In the literature, in a cross-sectional study whose delimited objective was to identify the prevalence and factors associated with postoperative complications at the surgical site in bariatric surgery, the sample consisted of 197 patients. The results showed that females were the most prevalent (n=152; 77.2%) and the age group up to 45 years (n=162, 82.2%) was the most operated. Of the participants, 30 had postoperative complications, totaling 45 (the participant could have more than one complication). The complications identified were: seroma (n=14, 31.1%), incisional hernia (n=7, 15.5%), superficial dehiscence (n=5, 11.1%), deep dehiscence (n=5, 11.1%), hematoma (n=4, 8.9%), infection (n=3, 6.7%), fistula (n=3, 6.7%), hemorrhage (n=2, 4, 4%), ischemia (n=1, 2.2%) and skin lesion (n=1, 2.2%). Of the factors associated with the outcomes, the authors highlighted the open approach (Odds ratio/OR=5.35), insertion of drains (OR=4.48) and a postoperative period of more than three days of hospitalization (OR= 5.03)^27^.

Next, we present the primary studies, whose sample was submitted to bariatric surgery through different surgical approaches^4^ ^,^ ^20^ ^-^ ^23^. In the prospective cohort study, the sample consisted of 2,012 participants, of which 356 (17.7%) underwent open surgery and 1,656 (82.3%) underwent laparoscopic surgery. The overall rate of SSI was 1.4% (28/2012), 1.6% (26/1656) laparoscopically and 0.6% (2/356) by open surgery, although this difference was not significant. (p=0.14). The results also showed that patients who received vancomycin as the only prophylactic antimicrobial were nine times more likely to develop SSI than patients who received other prophylaxis regimens (Relative Risk=9.4)^20^.

In the case-control study, patients underwent Roux-en-Y gastric bypass (n=751), with 701 laparoscopic (94%) and 46 open (6.1%) surgical procedures. The overall rate of SSI was 12% (n=91), with 71.4% of cases (n=65) being classified as superficial SSI, 19.8% (n=18) deep infections and 9.9% (n=8) of organ/space^21^.

In the cohort study, patients underwent gastric bypass (n=47,660), with 97% (n=46,231) laparoscopically, 2.3% (n=1,093) open surgeries, and 0.7% (n=1,093) =336) were laparoscopic procedures converted to open surgery. In this research, the authors investigated the risk of complications and mortality in relation to age. The overall deep infection/abscess rate was 9%. Superficial wound infection occurred in 1% of all patients in the cohort. The risk of developing superficial wound infection significantly increased in patients aged ≥60 years (OR=2.02) and 60-64 years (OR=2.14)^22^.

In the prospective cohort study of the investigated sample (n=1,596), 20.9% of patients (n=334) underwent open surgery and 79.1% (n=1,262) underwent laparoscopic surgery. Superficial SSI occurred in 16 patients (1%) and intra-abdominal infection in 15 (0.9%). Superficial SSI rates were 3% in the open approach group and 0.5% in the laparoscopic group (p < 0.05). The results showed a correlation of superficial SSI with the investigated variables (BMI and diabetes mellitus). Thus, the increase in BMI ranges was related to a higher occurrence of infection. In the sample, 2.2% (n=9) of patients with diabetes developed infection and 0.6% (n=7) of patients without this disease (p<0.05). It is noteworthy that in this study, the authors tested a care package (bundle) aimed at reducing SSI in bariatric surgery^23^.

In the retrospective study, the authors investigated the routine use of abdominal drain placement in bariatric surgery to assess complications associated with this practice and potential risk factors. Data were collected from the Metabolic and Bariatric Surgery Accreditation and Quality Improvement Program (MBSAQIP) database. Patients (n=148,260) underwent sleeve gastrectomy or Roux-en-Y gastric bypass (laparoscopic or robotic). The drain was used in 23,190 cases (15.6%) and not applied in 125,070 (84.4%). In the research, despite the SSI being an evaluated outcome, the researchers did not describe the occurrence/incidence of this complication. However, the results showed that drain placement during surgery was associated with an increased probability of superficial SSI (OR=1.57), deep incisional SSI (OR=2.04 and organ/space SSI (OR=1.8) ^4^.

Considering the results of the mentioned primary studies^4^ ^,^ ^20^ ^-^ ^23^, SSI rates ranged from 0.9% to 12%.

There is evidence in the literature about SSI and the different surgical approaches. In a case-control study, the authors compared different outcomes (eg, mortality, need for transfusion, presence of drain, among others) in bariatric surgery using robotic or laparoscopic approaches. The types of surgeries analyzed were Roux-en-Y gastric bypass (n=77,991, with 5,817 robotic procedures) and sleeve gastrectomy (n=189,503, with 12,912 robotic procedures). The MBSAQIP database was used for data collection in the period 2015-2016. In the robotic approach, the results showed lower occurrences of superficial SSI in patients undergoing Roux-en-Y gastric bypass (p=0.0003) and organ/space in sleeve gastrectomy (p=0.0002)^28^.

In contrast, in a retrospective study, the authors compared the perioperative results of sleeve gastrectomy by robotic or laparoscopic approaches in patients with a BMI≥50 kg/m^2^. The sample consisted of 61,493 patients (4,685 robotic procedures and 56,808 laparoscopic procedures). The MBSAQIP database was also used for data collection in the period 2015-2017. The group of patients undergoing robotic surgery had a longer duration of surgery (mean of 102.4 versus 74.7 minutes, p<0.001) and length of hospital stay (mean of 1.79 versus 1.66 days, p<0.01). In the multivariate analysis, the robotic approach was an independent risk factor for organ/space SSI^29^.

In a retrospective study with the participation of 772 patients who underwent Roux-en-Y gastric bypass or sleeve gastrectomy in a tertiary-level hospital in the United States of America, the objective was to identify risk factors for early complications in bariatric surgery. The results showed that open surgery was associated with the occurrence of superficial and organ/space SSI, in comparison with the laparoscopic approach^30^.

Antibiotic prophylaxis^20^ ^-^ ^21^ was the only risk factor investigated in at least two studies included in the review and delimited in this grouping.

In a literature review, the delimited objective was to evaluate research on the use of antibiotic prophylaxis in patients undergoing bariatric surgery to prevent SSI, with 16 studies being included in the sample (randomized clinical trial and observational studies). Thus, based on the results of the analyzed studies, the authors stated that cefazolin is the most effective antimicrobial, studied and used in bariatric surgery, and the administration of this drug, before anesthetic induction, should be considered as the first choice for antibiotic prophylaxis. However, dosage is still a problem, with the use of several different regimens with different reports of results^31^.

Due to the current discussion about the use of drains in bariatric surgery, in the present review, only in one study, the device was the risk factor investigated^4^.

In the literature, in a comparative study, the delimited objective was to describe the results achieved after the implementation of a bundle to reduce the occurrence of SSI and to identify the risk factors for this type of infection in bariatric surgery. Of the total sample (n=2,022), 53.6% patients underwent Roux-en-Y gastric bypass, 34.8% sleeve gastrectomy, 1.4% laparoscopic adjusted gastric band, 0.4% duodenal interruption, 7.9% were cases of revisions and 1.9% of procedures were categorized in “other category”. All surgical approaches (open, laparoscopic or robotic) were included in the sample. Of the participants, 1,977 (97.8%) had no infectious complications and 45 (2.2%) developed SSIs. Before the implementation of the care package, the SSI rate was 5.1%, with a significant reduction to 1.5% (after implementation of the bundle). The predictive factors for SSI were diabetes mellitus; the placement of a drain in the intraoperative period; the number of medications for hypertension prior to surgery and the open surgical approach^32^.

In a retrospective study, the authors defined the objective of identifying common preoperative characteristics that could have led to drain placement, surgical variables associated with drain placement, and differences in postoperative complications in patients who received such a device in bariatric surgery. Data were also collected from the MBSAQIP database, in the period 2015-2017. During this period, 388,239 bariatric surgeries were performed without drains and 100,221 were performed with drains. Surgical procedures included in the study were sleeve gastrectomy, Roux-en-Y gastric bypass and revisions. The results showed that 29% of patients undergoing gastric bypass had a drain placed, but only 16.7% of patients undergoing sleeve gastrectomy. The percentage of participants with a drain dropped from 33.1% to 24.6% in the study period and from 20.3% to 13.6%, respectively. The authors concluded that despite the reduction in the use of drains in bariatric surgery, the use of this device is still very common^33^.

Regarding the limitations of the integrative review, the inclusion of primary published studies was limited, that is, the gray literature was not considered, and there were restrictions on languages and period. Data analysis and synthesis were performed in a descriptive manner. Thus, combining data from different types of studies is a challenging process that can lead to bias in the elaboration of review results.

On the other hand, the search for primary studies was carried out in the main health and nursing databases. In addition, to assess the methodological quality of the research, the authors used tools developed by the Joanna Brigss Institute. This step reinforces the rigor in conducting the knowledge synthesis method.

## Conclusion

Antibiotic prophylaxis, female gender, high Body Mass Index and perioperative hyperglycemia were the main risk factors for the development of surgical site infection in bariatric surgery.

Surgical site infection rates ranged from 0.4% to 7.6%, considering the results of primary studies (n=6), in which patients underwent laparoscopic surgery. In primary studies (n=5), with participants undergoing procedures with different approaches (open, laparoscopic or robotic), surgical site infection rates ranged from 0.9% to 12%.

Conducting the integrative review generated a body of evidence that reinforces the importance of implementing effective measures for the prevention and control of surgical site infection by health professionals after bariatric surgery, promoting improved care and patient safety in the perioperative period.
